# Miltefosine Against *Scedosporium* and *Lomentospora* Species: Antifungal Activity and Its Effects on Fungal Cells

**DOI:** 10.3389/fcimb.2021.698662

**Published:** 2021-07-23

**Authors:** Rodrigo Rollin-Pinheiro, Yuri de Castro Almeida, Victor Pereira Rochetti, Mariana Ingrid Dutra da Silva Xisto, Luana Pereira Borba-Santos, Sonia Rozental, Eliana Barreto-Bergter

**Affiliations:** ^1^ Departamento de Microbiologia Geral, Instituto de Microbiologia Paulo de Góes, Universidade Federal do Rio de Janeiro, Rio de Janeiro, Brazil; ^2^ Programa de Biologia Celular e Parasitologia, Instituto de Biofísica Carlos Chagas Filho, Universidade Federal do Rio de Janeiro, Rio de Janeiro, Brazil

**Keywords:** miltefosine, *Scedosporium*, plasma membrane, antifungal drugs, drug repurposing, biofilm, fungal growth, lipid rafts

## Abstract

*Scedosporium* and *Lomentospora* species are filamentous fungi responsible for a wide range of infections in humans and are frequently associated with cystic fibrosis and immunocompromising conditions. Because they are usually resistant to many antifungal drugs available in clinical settings, studies of alternative targets in fungal cells and therapeutic approaches are necessary. In the present work, we evaluated the *in vitro* antifungal activity of miltefosine against *Scedosporium* and *Lomentospora* species and how this phospholipid analogue affects the fungal cell. Miltefosine inhibited different *Scedosporium* and *Lomentospora* species at 2–4 µg/ml and reduced biofilm formation. The loss of membrane integrity in *Scedosporium aurantiacum* caused by miltefosine was demonstrated by leakage of intracellular components and lipid raft disorganisation. The exogenous addition of glucosylceramide decreased the inhibitory activity of miltefosine. Reactive oxygen species production and mitochondrial activity were also affected by miltefosine, as well as the susceptibility to fluconazole, caspofungin and myoricin. The data obtained in the present study contribute to clarify the dynamics of the interaction between miltefosine and *Scedosporium* and *Lomentospora* cells, highlighting its potential use as new antifungal drug in the future.

## Introduction

*Scedosporium* and *Lomentospora* species are ubiquitous filamentous fungi known to be emergent pathogens that cause localised to disseminated infections with a broad range of clinical manifestations. Immunocompromised individuals, such as organ transplant recipients, HIV/AIDS patients and cystic fibrosis patients, are at greater risk of developing invasive infections with high mortality rates (Cortez et al., 2008; Luplertlop, 2018; Engel et al., 2019). In patients with cystic fibrosis, *Scedosporium* and *Lomentospora* species are frequently associated with colonisation of the lungs and are considered the second most frequent fungi that cause infection after *Aspergillus* species (Engel et al., 2019). In addition, infections caused by *Scedosporium* and *Lomentospora* species account for 25% of non-*Aspergillus* mould infections in transplant recipients (Husain et al., 2005). In this context, *Scedosporium aurantiacum* is a clinically relevant species related to severe disseminated infections as well as to the development of brain abscesses (Heath et al., 2009; Nakamura et al., 2013). In experimental models, *S. aurantiacum* induced 80% mortality in immunocompetent mice, an effect that could be correlated to its capacity to germinate rapidly, to resist oxidative stress and to form robust biofilm on various types of surfaces, such as central venous catheters and cell cultures (Gilgado et al., 2009; Mello et al., 2016; Rollin-Pinheiro et al., 2017; Staerck et al., 2018).

*S. aurantiacum* and other *Scedosporium* and *Lomentospora* species present intrinsic resistance to a wide variety of antifungals, such as amphotericin B, itraconazole, caspofungin and micafungin (Lackner et al., 2012), with voriconazole being the first choice for drug therapy of scedosporiosis (Tortorano et al., 2014). However, *in vitro* tests have demonstrated that their biofilms are significantly less susceptible to antifungal drugs, including voriconazole (Rollin-Pinheiro et al., 2017). Because treatment options for scedosporiosis are restricted, new strategies are necessary.

Glycoconjugates from the fungal surface are essential for fungal viability, morphogenesis and pathogenesis. Therefore, these molecules represent important new targets for antifungal therapy (Gow et al., 2017; Rollin-Pinheiro et al., 2020). In this context, glucosylceramide (GlcCer) is the main sphingolipid present in the fungal cell wall and membrane (Barreto-Bergter et al., 2011). GlcCer is a determinant for the growth and virulence of *Cryptococcus neoformans* and *Candida albicans* (Rittershaus et al., 2006; Oura and Kajiwara, 2008). In filamentous fungi such as *Aspergillus nidulans*, *Colletotrichum gloeosporioides* and *Scedosporium apiospermum*, it has been shown that GlcCer plays an important role in hyphal growth (da Silva et al., 2004; Rollin-Pinheiro et al., 2014; Fernandes et al., 2016). Additionally, monoclonal antibodies against GlcCer presented a synergistic effect with itraconazole in *S. apiospermum* and the sphingolipid synthesis inhibitor myriocin reduced biofilm formation and membrane integrity of *Scedosporium boydii*, highlighting GlcCer and other sphingolipids as potential targets for alternative antifungal treatment (Rollin-Pinheiro et al., 2014; Rollin-Pinheiro et al., 2019).

Miltefosine is a phospholipid analogue belonging to the alkylphosphocholine class. Initially developed as an antitumor agent, the drug is also active against protozoan species, including *Leishmania* spp., *Trypanosoma cruzi*, *Trichomonas vaginalis* and *Plasmodium falciparum* (Urbina, 2006; Dorlo et al., 2012a; Dorlo T. P. C. et al., 2012). It was the first oral drug licensed for the treatment of visceral and cutaneous leishmaniasis, being used in India and Colombia (Calogeropoulou et al., 2008). In Brazil, it has been commercialised and is used for *Leishmania* infections in dogs and has been tested against human leishmaniosis. Studies have demonstrated that the mode of action of miltefosine in human cancer cells and *Leishmania* is linked to apoptosis and interference in lipid-dependent signalling pathways (Dorlo T. P. C. et al., 2012).

It has already been shown that miltefosine has *in vitro* antifungal activity against several medically important fungi such as dermatophytes, *Cryptococcus* spp., *Candida* spp., *Sporothrix* spp., *Paracoccidioides* spp., *Histoplasma capsulatum*, *Coccidioides posadasii*, *Rhizopus* spp., *Aspergillus* spp., *Fusarium* spp. and *Scedosporium* spp. (Widmer et al., 2006; Tong et al., 2007; Vila et al., 2013; Imbert et al., 2014; Borba-Santos et al., 2015; Brilhante et al., 2015; Compain et al., 2015; Vila et al., 2016; Rossi et al., 2017). Miltefosine showed *in vitro* activity against *S. apiospermum*, *S. aurantiacum* and *Lomentospora prolificans*, a species closed related to the *Scedosporium* group (Compain et al., 2015). The authors of two studies have reported the successful use of miltefosine in combination with voriconazole and terbinafine for the treatment of *L. prolificans* infections (Kesson et al., 2009; Trubiano et al., 2014). These preliminary works indicate the potential of miltefosine as an alternative option for drug therapy in *Scedosporium* and *Lomentospora* species infections.

Little is known about the mechanism of action and the anti-biofilm properties of miltefosine in *Scedosporium* and *Lomentospora* species. Therefore, this work aimed to evaluate the antifungal activity of miltefosine on different *Scedosporium* and *Lomentospora* species, as well as its effects on fungal growth, biofilm formation, cell membrane integrity, mitochondrial activity and reactive oxygen species (ROS) production.

## Materials and Methods

### Strains and Growth Conditions

*Scedosporium aurantiacum* CBS 136046, *Scedosporium boydii* CBS 120157, *Scedosporium apiospermum* CBS 117407 and *Scedosporium dehoogii* CBS 117406 were kindly provided by Sybren De Hoog, from the Westerdijk Fungal Biodiversity Institute, Utrecht, the Netherlands. *Lomentospora prolificans* FMR 3569 was kindly provided by Dr J. Guarro, Unitat de Microbiologia, Facultat de Medicina e Institut d`Estudis Avançats, Réus, Spain. All fungi were maintained in modified Sabouraud medium (0.5% yeast extract, 1% peptone and 2% glucose monohydrate). To obtain conidia, cells were grown on plates containing modified Sabouraud agar medium for 7 days at room temperature. After that, the surface of the medium was washed with sterile phosphate-buffered saline (PBS, pH 7.2), and the conidia were removed with the aid of a sterile spatula. The cell suspension was filtered and later centrifuged to be used in the experiments.

### Antifungal Susceptibility Testing

The susceptibility of *Scedosporium* and *Lomentospora* species to miltefosine was determined by the broth microdilution method, according to EUCAST protocols, with modifications (Taj-Aldeen et al., 2016). Miltefosine (Cayman Chemical Co., Ann Arbor, MI, USA) was diluted in dimethyl sulphoxide:ethanol (1:1) to obtain stock solutions of 6400 μg/ml and maintained at -20°C. Briefly, a standardised suspension of conidia (2 × 10^5^/ml) was incubated in 96-well plates containing RPMI 1640 medium (Sigma-Aldrich, St. Louis, MO, USA) supplemented with 2% glucose and buffered with 3-(*N*-morpholino)propanesulphonic acid (MOPS) 0.165 mol/l, pH 7.0 (from here on referred to as ‘supplemented RPMI’). Miltefosine was serially diluted (64–0.062 μg/ml) and added to the microplates. After 72 h of incubation at 37°C, the cell viability was assessed using the XTT-reduction assay (Rollin-Pinheiro et al., 2019). The minimum inhibitory concentration (MIC) of miltefosine was defined as the lowest concentration that inhibits 90% of fungal growth.

Voriconazole was used as a standard in the experiments because it is the drug of choice for the treatment of scedosporiosis. Voriconazole was serially diluted (100–0.097 μg/ml) and used as described above.

### Kinetics of Fungal Growth

*Scedosporium* and *Lomentospora* conidia (1 × 10^5^) were incubated in 96-well microplates containing supplemented RPMI. Miltefosine at different concentrations (1, 2, 4 and 8 μg/ml, corresponding to 0.5× MIC, MIC and 2× MIC) was added to the microplates prior to incubation at 37°C. Cells without miltefosine were used as positive control. The growth kinetics was evaluated according to (Rollin-Pinheiro et al., 2019). Fungal growth was analysed according to the optical density (OD) of the samples. The OD was measured at 660 nm every 2 h up to a total 24 h of incubation using the Cytation 5 Imaging Reader (BioTek, Winooski, VT, USA).

### Germination

Aiming to observe fungal germination, *Scedosporium* and *Lomentospora* conidia (1 × 10^5^) were incubated in 24-well microplates containing supplemented RPMI in the presence of miltefosine at the MIC for each strain. Cells without miltefosine were used as a positive control. After 0, 6, 12, 18 and 24 h of incubation at 37°C, the cells were photographed using the Cytation 5 Imaging Reader (BioTek).

### Biofilm Formation and the Preformed Biofilm Assay

Biofilm formation was analysed according to (Rollin-Pinheiro et al., 2017). Briefly, a standardised suspension of *Scedosporium* and *Lomentospora* conidia (1 × 10^7^/ml) was added to each well of a microplate and incubated for 1.5 h at 37°C for the adhesion step. After that, the supernatant containing non-adherent cells was removed and RPMI 1640 medium supplemented with MOPS, 2% glucose and 20% foetal bovine serum (FBS, Gibco, MA, USA) was added in the absence (positive control) or presence of miltefosine (1–32 μg/ml). Adherent cells were then incubated for 24 h at 37°C. For the preformed biofilm assay, cells were cultured to form biofilm as described above in the absence of miltefosine. After 24 h of biofilm formation, the supernatant was removed and supplemented RPMI was added in the absence (positive control) or presence of miltefosine (varying from 1–32 μg/ml). An additional incubation of 24 h at 37°C was performed to evaluate miltefosine activity. Both biofilm formation and preformed biofilms were evaluated through three parameters as previously described (Mello et al., 2016; Mello et al., 2018; Rollin-Pinheiro et al., 2019). Crystal violet, safranin and XTT were used to analyse the overall biomass, extracellular matrix and metabolic activity, respectively.

### Scanning Electron Microscopy

*S. aurantiacum* cells were grown in supplemented RPMI in the absence or the presence of miltefosine (1, 2 and 4 μg/ml, corresponding to 0.25× MIC, 0.5× MIC and MIC), with orbital agitation (150 rpm) for 48 h. Cells were centrifuged, washed in sterile PBS and processed according to the following steps: (i) fixation in 2.5% glutaraldehyde and 4% formaldehyde, in 0.1 M cacodylate buffer, for 1 h at room temperature; (ii) three washes in 0.1 M cacodylate buffer; (iii) adhesion to poly-l-lysine-coated glass coverslips; (iv) post-fixation in 1% osmium tetroxide in 0.1 M cacodylate buffer containing 1.25% potassium ferrocyanide and 5 mM CaCl_2_ for 30 min; (v) dehydration in a graded ethanol series (30%–100%); (vi) critical point drying in CO_2_; and (vii) coating with gold. Images were obtained with TESCAN VEGA3 scanning electron microscope (Tescan Analytics, Provence, France) and processed using Photoshop software (Adobe, CA, USA).

### Evaluation of Oxidative Stress

ROS production by *S. aurantiacum* cells was evaluated according to (Spadari et al., 2018). Briefly, conidia (1 × 10^5^) were grown in supplemented RPMI in the absence or the presence of different concentrations of miltefosine (4 and 8 μg/ml, corresponding to MIC and 2× MIC, respectively) for 6 h (incubation time when fungal viability starts to be affected) at 37°C. After incubation, the cells were centrifuged, washed with sterile PBS and labelled with 5 μM of the fluorescent dye 2’,7’-dichlorofluorescein diacetate (DCFH-DA) (Sigma-Aldrich) for 30 min at room temperature in the dark. After treatment with DCFH-DA, the samples were washed three times to remove residual dye and 1 × 10^5^ cells were suspended in PBS. The fluorescence intensity was measured using the SpectraMax 340 microplate reader (Molecular Devices, CA, USA) at 492 nm (excitation) and 517 nm (emission)

### Evaluation of the Mitochondrial Membrane Potential

The mitochondrial membrane potential (ΔΨm) of *S. aurantiacum* cells after miltefosine exposure was determined using the fluorescent probe JC-1 (Thermo Fisher Scientific, MA, USA) according to (Garcia et al., 2018). After miltefosine treatment with 4 and 8 μg/ml for 6 h, the cells (1 × 10^5^) were centrifuged, washed with sterile PBS and labelled with 5 μM of JC-1 for 30 min at 37°C in the dark. Then, the samples were washed three times to remove residual dye and 1 × 10^5^ cells were suspended in PBS. The fluorescence intensity was measured using the SpectraMax 340 microplate reader (Molecular Devices) at the following conditions: excitation at 475 nm and emission at 529 nm (green fluorescence) or 590 nm (red fluorescence). The ratio of red to green fluorescence intensity was calculated.

### Filipin Staining

*S. aurantiacum* cells were stained with filipin according to (Rollin-Pinheiro et al., 2019). Briefly, conidia (1 × 10^5^) were grown in 24-well plates containing supplemented RPMI in the absence or the presence of 2 μg/ml (0.5× MIC) of miltefosine for 6 h at 37°C. Then, the germinated conidia were washed three times with PBS and stained with 50 μg/ml of filipin (Sigma-Aldrich, F9765) for 2 h at room temperature in the dark. After washing three times with PBS, the cells were observed under a fluorescence microscope (Axioplan Imager 2, Carl Zeiss, Oberkochen, Germany).

### Susceptibility to Membrane and Cell Wall Stressors

The susceptibility to surface stressors was analysed according to (Rollin-Pinheiro et al., 2019). Conidia of *S. aurantiacum* (1 × 10^5^) were grown in 96-well plates containing supplemented RPMI in the absence (positive control) and in the presence of sub-inhibitory concentrations of miltefosine (1 and 2 μg/ml, corresponding to 0.25× MIC and 0.5× MIC) for 24 h at 37°C. Then, the supernatant was removed from the microplates and sodium dodecyl sulphate (SDS, 90 μg/ml) and calcofluor white (10 μg/ml; Sigma-Aldrich) were added. After another 24-h incubation, the cell viability was measured by using the XTT-reduction assay and readings were captured using a spectrophotometer (Bio-Rad, Hercules, CA, USA) at 490 nm.

### Evaluation of Cell Membrane Permeability

The cell membrane permeability was determined by measuring the release of DNA and protein to the culture supernatant (Spadari et al., 2018). Conidia of *S. aurantiacum* (1 × 10^5^) were grown in supplemented RPMI in the absence (positive control) or presence of miltefosine (2, 4, 8 and 16 μg/ml, corresponding to 0.5× MIC, MIC, 2× MIC and 4× MIC, respectively) for 6, 12, 18 and 24 h at 37°C. After each incubation time, the cells were pelleted and the supernatant analysed using a NanoDrop 2000 spectrophotometer (Thermo Fisher Scientific) to quantify free DNA (260 nm) and proteins (280 nm). A sterile culture medium sample was used as a negative control.

Fluorescence microscopy was also used to evaluate cell membrane permeability by staining cells with Sytox Green (Thermo Fisher Scientific) and calcofluor white. Conidia of *S. aurantiacum* (1 × 10^5^) were grown in supplemented RPMI in the absence (positive control) or presence of 0.5× MIC of miltefosine, as well as in the presence of SDS (120 μg/ml) as a positive control. The cells were incubated for 24 h at 37°C, then washed three times. Next, the samples were stained with Sytox Green (50 μg/ml) and calcofluor white (50 μg/ml) for 1 h at room temperature protected from the light. After washing three times, the samples were observed using a fluorescence microscope (Axioplan Imager 2, Carl Zeiss). The emission/excitation wavelengths used were 523 nm/504 nm for Sytox Green and 475nm/380 nm for calcofluor white.

### Susceptibility in the Presence of Exogenous GlcCer

*S. aurantiacum* susceptibility to miltefosine in the presence of exogenous GlcCer was determined according to EUCAST protocols, with modifications (Taj-Aldeen et al., 2016; Spadari et al., 2018). *S. aurantiacum* (1 × 10^5^ conidia) susceptibility to miltefosine was evaluated as described in section 2.2, but in the presence of exogenous GlcCer (previously purified from the same strain) (Caneppa et al., 2019) at concentrations of 50 and 100 μg/ml. After 72-h incubation at 37°C, the cell viability was determined by the XTT-reduction assay, with the MIC of miltefosine defined as the lowest concentration that inhibits 90% of fungal growth.

### Antifungal Drug Synergy Assay

Synergistic interactions were detected by the checkerboard method according to EUCAST guidelines (Subcommittee on Antifungal Susceptibility Testing of the, E.E.C.f.A.S.T., 2008). *S. aurantiacum* conidia (1 × 10^5^) were grown in 96-well plates containing supplemented RPMI in the presence of miltefosine (0.125–8 μg/ml) combined with myriocin (0.5–64 μg/ml) (a sphingolipid inhibitor that displays antifungal activity) (Rollin-Pinheiro et al., 2019), fluconazole (1.57–100 μg/ml) or caspofungin (1.57–100 μg/ml). After a 72-h incubation at 37°C, the MIC was evaluated at 600 nm and the cell viability was assessed by the XTT-reduction assay at 490 nm using a spectrophotometer (Bio-Rad). Interactions were determined by the fractional inhibitory concentration index (FICI) that was calculated using the following formula: (MIC combined/MIC drug A alone) + (MIC combined/MIC drug B alone). The results were classified as: synergistic effect, FICI of ≤ 0.5; no effect, FICI of > 0.5–4.0; antagonistic effect, FICI of > 4.0 (Odds, 2003).

### Citoxicity Assay

Cytotoxicity was analysed by neutral red (NR) assay with modifications (Borenfreund and Puerner, 1985). RAW 264.7 (murine monocytes/macrophages) cell monolayer was harvested with a cell scraper and viable cells were counted using the Trypan blue exclusion method. 2 x 10^5^ macrophages per well were seeded in 96-well plates containing Dulbecco’s Modified Eagle Medium (DMEM) with 10% FBS and incubated in a controlled atmosphere of 5% CO_2_ at 37°C for adhesion. Miltefosine was serial diluted in DMEM and cells were incubated at concentrations of 2.5, 5, 10, 20 and 40 µg/mL at 37°C, 5% CO_2_ for 24 h. Cells without miltefosine were used as control. Absorbance was determined in a spectrophotometer at 595 nm (SpectraMax^®^ i3x, Molecular Devices^®^, EUA).

### Statistical Analyses

All experiments were performed in triplicate, in three independent experimental sets. Statistical analyses were performed using GraphPad Prism version 5.00 for Windows (GraphPad Software, San Diego, CA, USA). The nonparametric Kruskal–Wallis one-way analysis of variance was used to compare the differences among the groups, and individual comparisons of the groups were performed using a Bonferroni post-test. The 90% or 95% confidence interval was determined in all experiments.

## Results

### Miltefosine Inhibits *Scedosporium* and *Lomentospora* Growth and Viability

The MICs of miltefosine were evaluated to determine its inhibitory potential against *Scedosporium* and *Lomentospora* species. The MIC was the lowest concentration able to inhibit 90% of fungal growth. The MIC observed for *S. aurantiacum*, *S. apiospermum*, *S. dehoogii* and *L. prolificans* was 4 μg/ml, whereas for *S. boydii* it was 2 μg/ml (**Table 1**). Voriconazole was used as a standard in the analyses because it is the first choice to treat these infections in clinical settings. The MICs for voriconazole were 1.25 μg/ml for *S. aurantiacum* and *S. apiospermum*, 0.31 μg/ml for *S. boydii*, 0.625 μg/ml for *S. dehoogii* and 20 μg/ml for *L. prolificans* (**Table 1**).

**Table 1:** MIC_90_, MFC and viability inhibition of miltefosine and voriconazole against *Scedosporium* and *Lomentospora* species.

	Miltefosine (μg/ml)		Voriconazole (μg/ml)	
	Growth inhibition (MIC_90_)	MFC	Growth inhibition (MIC_90_)	MFC
*Scedosporium aurantiacum*	4	4	1.25	1.25
*Scedosporium boydii*	2	2	0.31	0.62
*Scedosporium apiospermum*	4	4	1.25	2.5
*Scedosporium dehoogii*	4	4	0.625	0.625
*Lomentospora prolificans*	4	4	20	20

MIC_90_, Minimum inhibitory concentration that inhibits 90% of fungal growth.

MFC, minimum fungicidal concentration.

Experiments were performed in triplicate, in three independent experimental sets, to calculate the minimum inhibitory concentration (MIC).

Viability inhibition was evaluated using the XTT-reduction assay. For miltefosine, the MICs were also able to inhibit viability of all fungi. Regarding voriconazole, there were similar results for *S. aurantiacum*, *S. dehoogii* and *L. prolificans*, whereas the concentration needed to inhibit fungal viability increased from 0.31 to 0.625 μg/ml for *S. boydii* and from 1.25 to 2.5 μg/ml for *S. apiospermum* (**Table 1**).

### Evaluation of Germination and Proliferation of *Scedosporium* and *Lomentospora* Species in the Presence of Miltefosine

**Figure 1:**
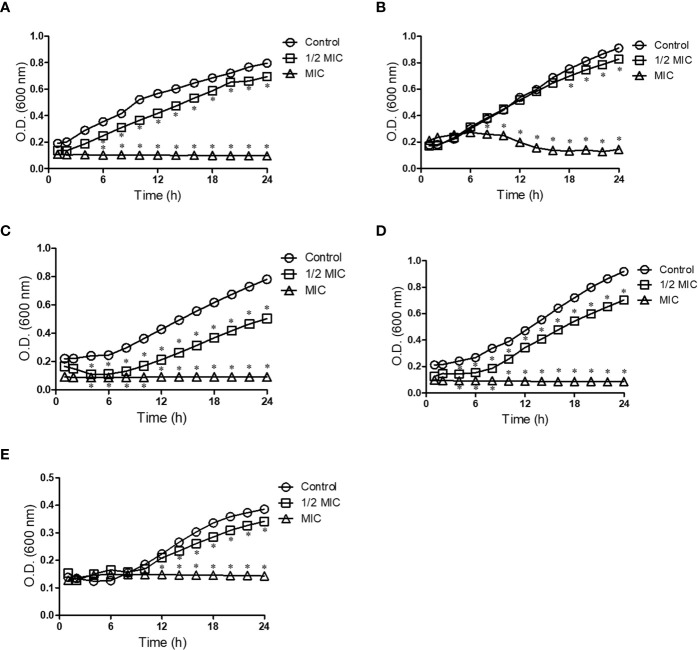
Kinetic growth of *Scedosporium aurantiacum* **(A)**, *Scedosporium boydii* **(B)**, *Scedosporium apiospermum* **(C)**, *Scedosporium dehoogii* **(D)** and *Lomentospora prolificans* **(E)**. Cells were incubated in the absence or the presence of different concentrations of miltefosine at 37°C for up to 24 h; the optical density was measured every 2 h. *p < 0.001.

Because *Scedosporium* and *Lomentospora* cells were susceptible to miltefosine, fungal growth kinetics was measured to observe its inhibitory effect over time at two concentrations: 0.5× MIC and MIC. The growth of control cells (untreated samples) was observed after 6 h of incubation and continued to increase over time (**Figure 1**). Whereas miltefosine at the MIC completely inhibited fungal growth, at 0.5× MIC this compound only partially reduced *S. apiospermum* and *S. dehoogii* growth and did not affect *S. aurantiacum*, *S. boydii* and *L. prolificans* growth (**Figure 1**).

**Figure 2:**
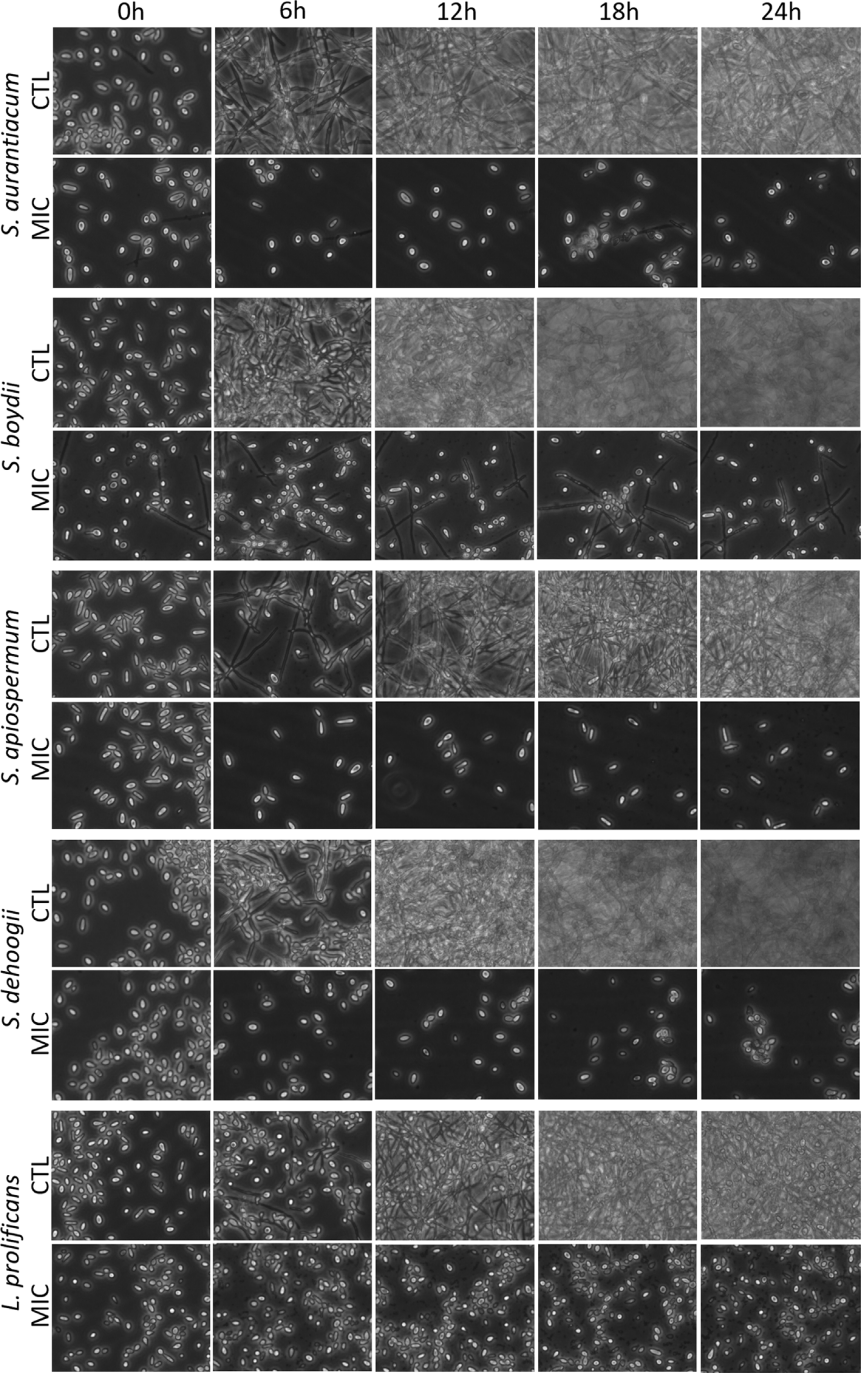
Fungal germination in the absence or the presence of the minimum inhibitory concentration (MIC) of miltefosine. Cells were incubated at 37°C for up to 24 h; pictures were recorded every 6 h.

To assess whether miltefosine alters fungal differentiation, conidia of all fungi were incubated in the absence (positive control) or presence of miltefosine (the MIC) for 0, 6, 12, 18 and 24 h. Compared with the control, miltefosine affected hyphal development (**Figure 2**). These data suggest that miltefosine affects *Scedosporium* and *Lomentospora* at early stages of growth.

### The Effect of Miltefosine on Fungal Biofilms

**Figure 3:**
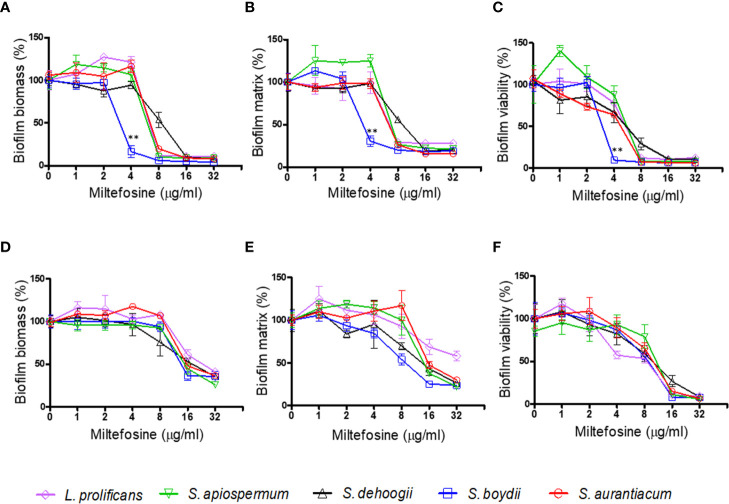
The effect of miltefosine on biofilm formation **(A–C)** and preformed biofilm **(D–F)** of *Scedosporium aurantiacum*, *Scedosporium boydii*, *Scedosporium apiospermum*, *Scedosporium dehoogii* and *Lomentospora prolificans*. The fungal biomass was measured by crystal violet **(A, D)**, the extracellular matrix was measured by safranin **(B, E)** and metabolic viability was evaluated by the XTT-reduction assay **(C, F)**. **p < 0.01.

*Scedosporium* and *Lomentospora* species have already been described to form biofilms, which is an important structure for fungal virulence (Mello et al., 2016; Rollin-Pinheiro et al., 2017). For this reason, the inhibition of *Scedosporium* and *Lomentospora* biofilm formation and the effect in preformed biofilms in presence of miltefosine were analysed. Miltefosine reduced biofilm formation, decreasing more than 50% the biomass and viability after exposure to 4 µg/ml (*S. boydii*), 8 µg/ml (*S. aurantiacum*, *S. apiospermum* and *L. prolificans*) and 16 µg/ml (*S. dehoogii*) (**Figures 3A–C**). These data corroborate the MIC analysis, because *S. boydii* was more susceptible compared with the other species in both the MIC and biofilm inhibition assays. Miltefosine also affected preformed biofilms: treatment of preformed biofilm led to a reduction of approximately 50%–60% of the biomass and viability for all fungi after incubation with 16 and 32 µg/ml of miltefosine (**Figures 3D–F**). These results suggest that miltefosine affects not only planktonic cells, but also *Scedosporium* and *Lomentospora* biofilm formation and mature biofilms.

**Figure 4:**
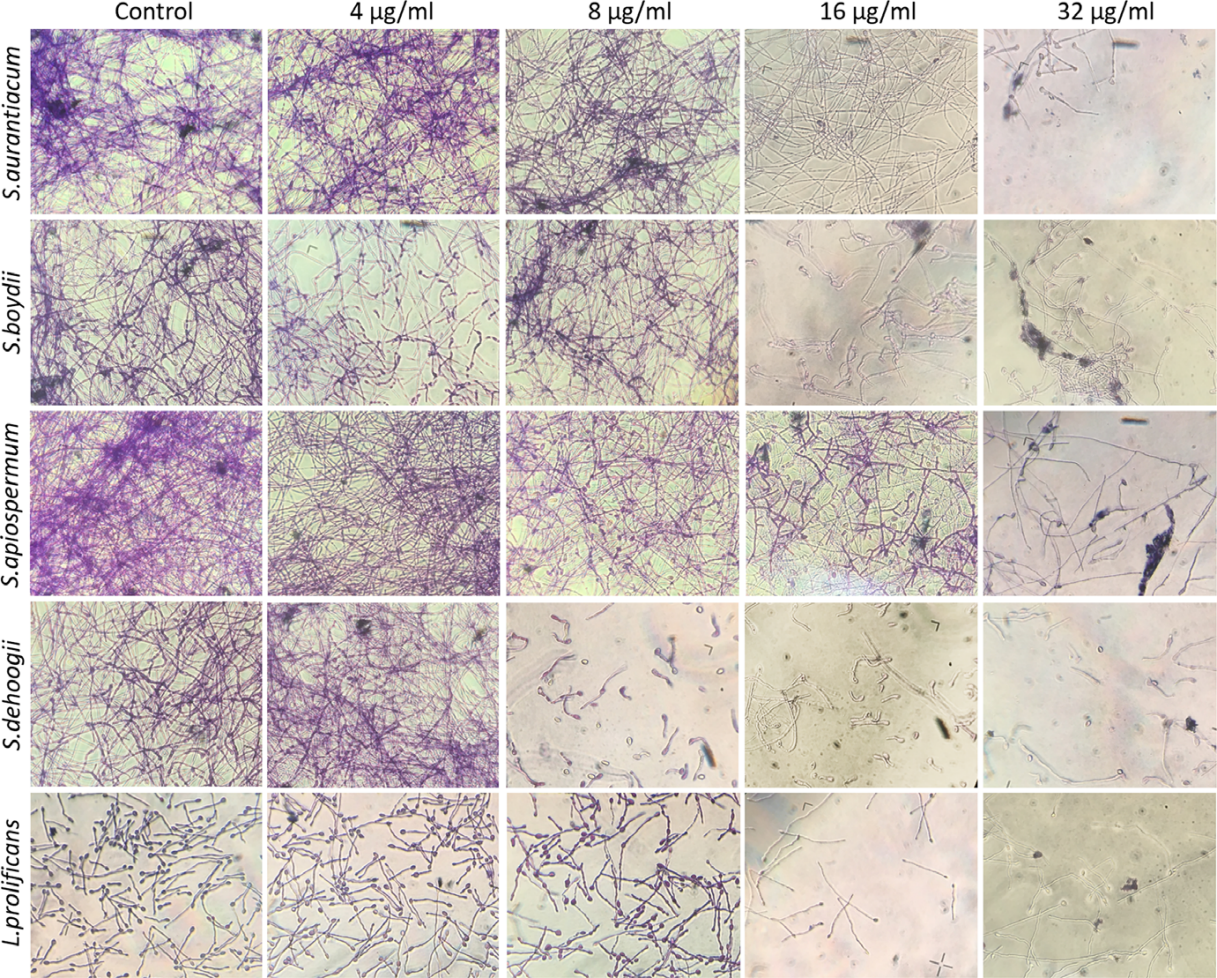
Preformed biomass of *Scedosporium aurantiacum*, *Scedosporium boydii*, *Scedosporium apiospermum*, *Scedosporium dehoogii* and *Lomentospora prolificans* observed using a light microscope. Cells were grown in RPMI 1640 at 37°C for 24 h to form a fungal biomass and then a new 24-h incubation was performed in the absence (control) or the presence of 4, 8, 16 or 32 μg/ml of miltefosine. The fungal biomass was then stained with crystal violet and observed using a light microscope.

Crystal violet staining showing fungal biomass is shown in **Figure 4**. There was reduced fungal growth, especially from 16 µg/ml of miltefosine.

### Alterations Caused by Miltefosine on *S. aurantiacum* Morphology

Aiming to understand how miltefosine affects *Scedosporium* and *Lomentospora* cells, *S. aurantiacum* was chosen as a representative species due to its high virulence and resistance to antifungal drugs, as has been reported in the literature (Gilgado et al., 2009; Harun et al., 2010). Hence, the subsequent analyses focused on *S. aurantiacum*.

**Figure 5:**
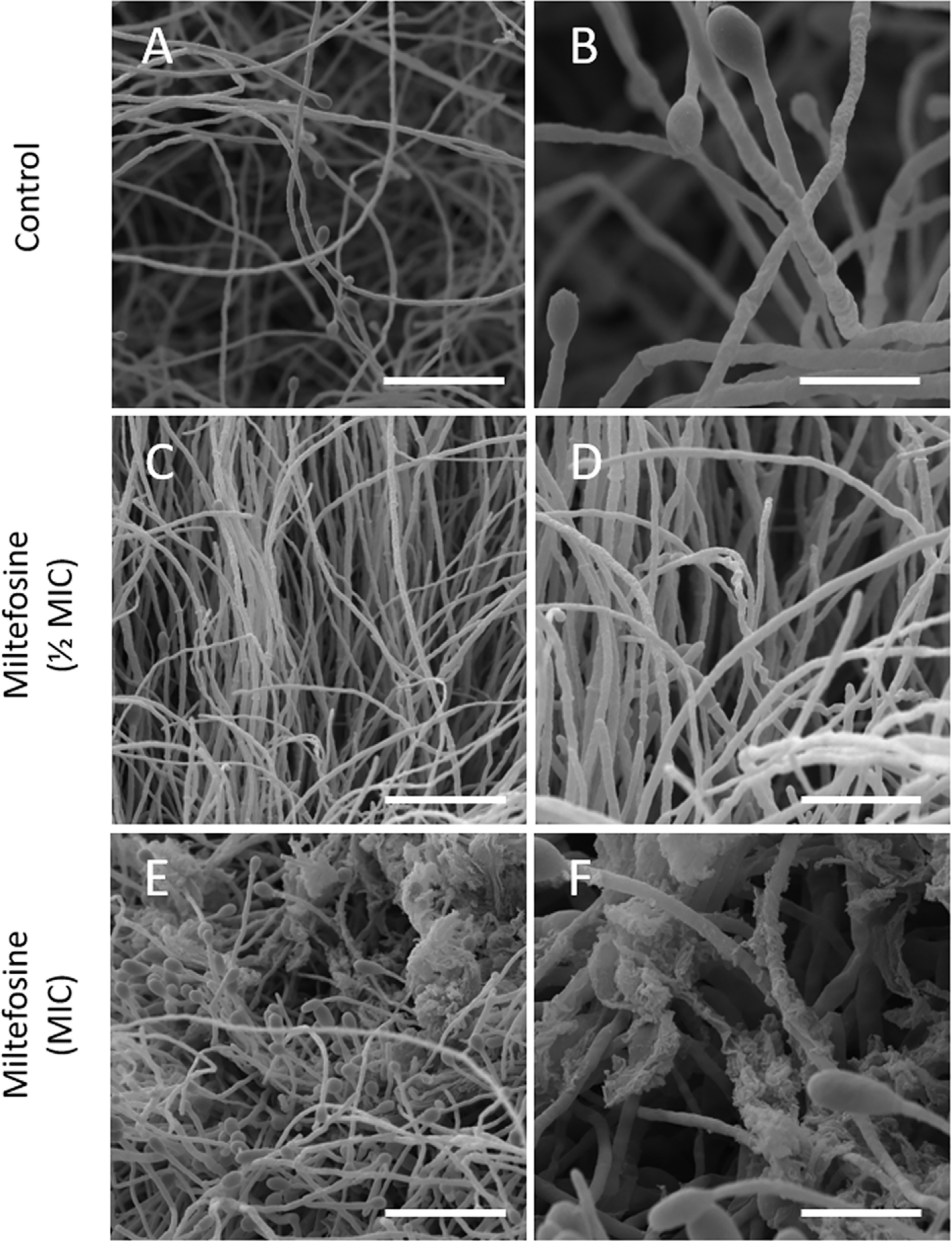
Scanning electron microscopy of *Scedosporium aurantiacum* cells grown for 48 h in the presence of miltefosine. Scale bars: 20 μm **(A, C, E)** and 10 μm **(B, D, F)**.

The effect of miltefosine on *S. aurantiacum* morphology was observed by scanning electron microscopy. *S. aurantiacum* control cells grown for 48 h in the absence of miltefosine showed a mycelium containing septate hyphae, conidiogenous cells and conidia (**Figures 5A, B**), whereas cells treated with sub-inhibitory concentrations of miltefosine (0.5× MIC) displayed some twisted and broken filaments (**Figures 5C, D**). When treated with the MIC of miltefosine, the *S. aurantiacum* surface was severely disrupted, evidencing intracellular leakage (**Figures 5E, F**).

### Evaluation of High Oxidative Stress and Mitochondrial Disturbances in Miltefosine-Treated Cells

**Figure 6:**
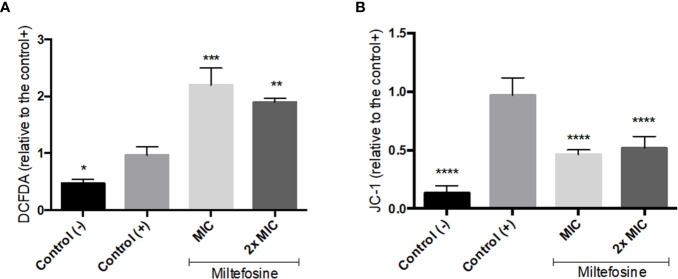
The effect of miltefosine on oxidative stress **(A)** and the mitochondrial membrane potential **(B)** in *Scedosporium aurantiacum* after 6 h of incubation. DCFH-DA and JC-1 fluorescent staining was used to measure reactive oxygen species (ROS) production and membrane polarisation, respectively. Control (-) represents cells in the absence of fluorescent stain. Control (+) represents cells stained with fluorescent DCFDA or JC-1, but without miltefosine treatment. *p < 0.05, **p < 0.01, ***p < 0.001, ****p < 0.0001.

To evaluate the effect of inhibitory concentrations of miltefosine in *S. aurantiacum*, the production of ROS was estimated using the fluorogenic dye DCFH-DA. The ROS production was approximately 2.0 and 1.9 fold higher compared with the untreated control cells in the presence of miltefosine at the MIC and 2× MIC, respectively (**Figure 6A**). In addition, mitochondrial disturbances were analysed using a fluorescent dye; there was a 50% decrease in the JC-1 red/green fluorescence ratio (**Figure 6B**). These results suggest that miltefosine induces high oxidative stress and interferes with the mitochondrial membrane potential (ΔΨm), as a result of mitochondrial dysfunction.

### The Effect of Miltefosine on Membrane Organisation

**Figure 7:**
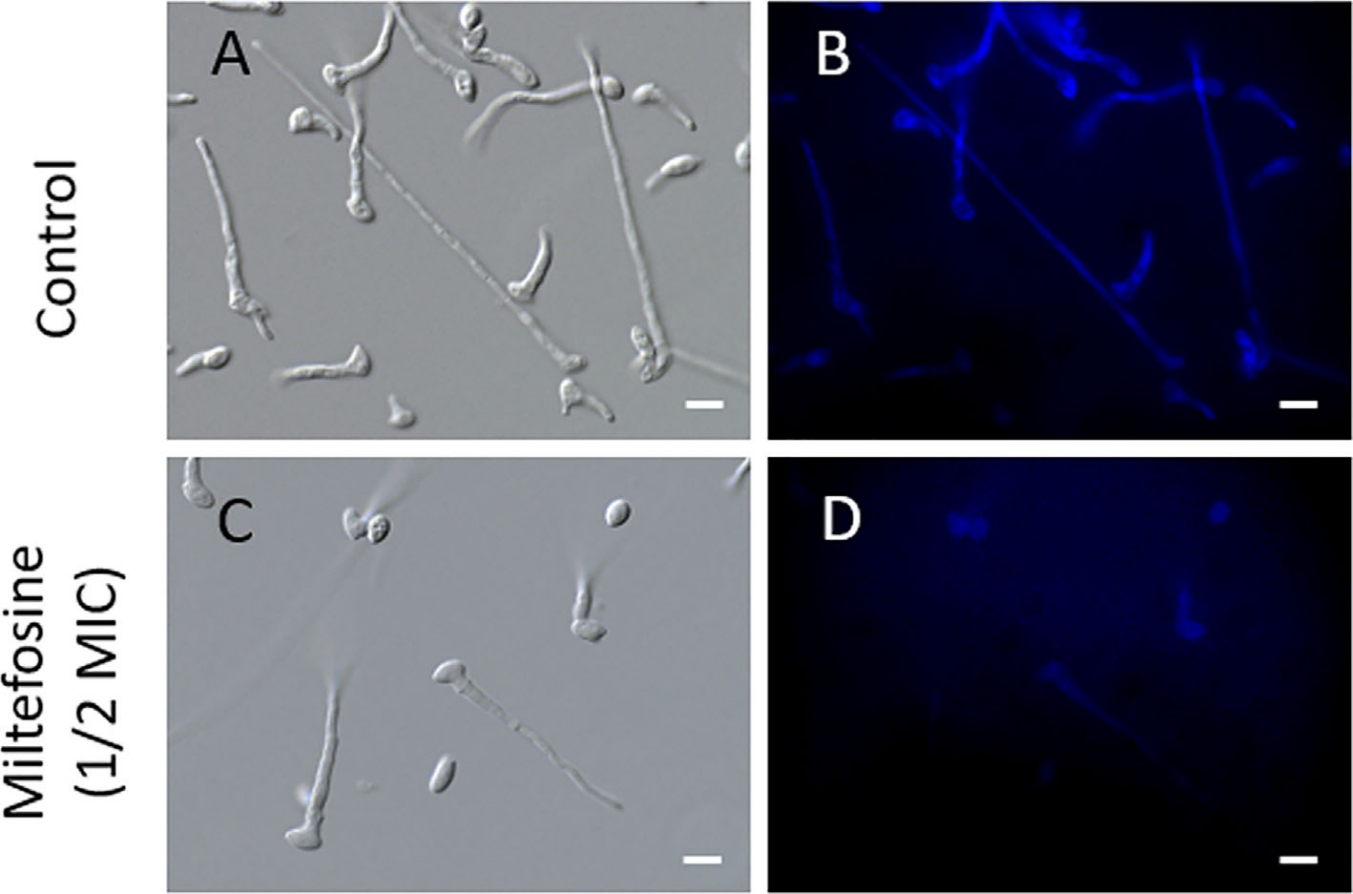
The effect of miltefosine on *Scedosporium aurantiacum* lipid rafts. Filipin staining was used to evaluate the presence of sterol, which is a common marker for lipid raft regions in fungal membranes. **(A, C)** represent differential interferential contrast microscopy. **(B, D)** represent fluorescence microscopy. The images show *S. aurantiacum* cells grown for 6 h at 37°C.

Due to the effect of miltefosine observed on *S. aurantiacum*, we evaluated how it acts on fungal cells. To observe changes in membrane organisation at early stages of fungal growth, *S. aurantiacum* conidia were incubated in the presence of a sub-inhibitory concentration (0.5× MIC) of miltefosine for 6 h and stained with filipin, a polyene commonly used to study the accumulation of sterols on fungal microdomains found in the plasma membrane. Compared with the control, the presence of miltefosine reduced filipin staining (**Figure 7**). These data suggest that the antifungal effect attributed to miltefosine at a sub-inhibitory concentration is related to the disorganisation of lipid rafts in the *S. aurantiacum* membrane, because there was decreased sterol staining in treated cells.

### Evaluation of *S. aurantiacum* Susceptibility to Membrane and Cell Wall Stressors After Treatment With Miltefosine

**Figure 8:**
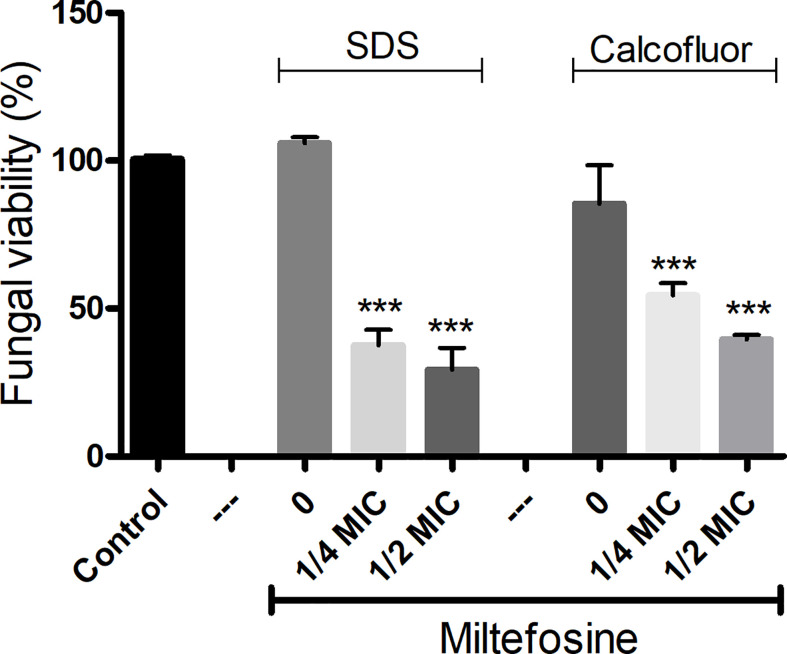
*Scedosporium aurantiacum* susceptibility to membrane (SDS) and cell wall (calcofluor white) stressors in the presence of miltefosine. The control represents fungal viability in the absence of stressors and miltefosine. SDS, sodium dodecyl sulphate. ***p < 0.001.

Because miltefosine had an effect on fungal lipid rafts at a sub-inhibitory concentration, we evaluated whether *S. aurantiacum* is more susceptible to surface stressors after treatment with miltefosine. Two different stressor agents were used: SDS, an anionic detergent and membrane stressor, and calcofluor white, a cell wall stressor. Cells treated with sub-inhibitory concentrations of miltefosine (0.25× MIC and 0.5× MIC) prior to incubation with SDS and calcofluor white showed an increase in susceptibility to non-inhibitory concentrations of both stressors compared with the control (**Figure 8**). SDS led to a > 50% reduction of fungal viability, whereas calcofluor white resulted in an approximately 50% decrease in *S. aurantiacum* viability (**Figure 8**). These results indicate that miltefosine affects the fungal plasma membrane and cell wall physiology.

### Miltefosine Seems to Increase Cell Membrane Permeability

**Figure 9:**
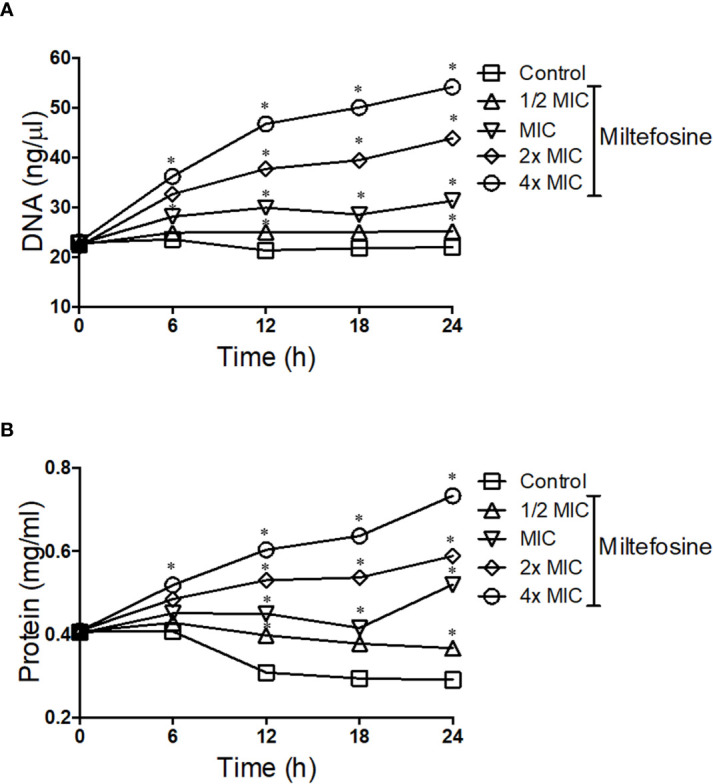
The kinetics of DNA **(A)** and protein **(B)** release by *Scedosporium aurantiacum* cells in the presence of different concentrations of miltefosine. *S. aurantiacum* cells were centrifuged after 0, 6, 12, 18 and 24 h of growth, and DNA and protein content were measured in the supernatant using NanoDrop (Thermo Fisher Scientific). *p < 0.001.

Due to the alterations caused by miltefosine in the plasma membrane, fungal permeability was analysed in more detail. *S. aurantiacum* was incubated with different concentrations of miltefosine (0.5× MIC, MIC, 2× MIC and 4× MIC) and the presence of DNA and protein in the supernatant of the fungal culture was measured every 6 h of incubation, until 24 h. Increasing levels of free DNA were observed in *S. aurantiacum* supernatant after growth in the presence of 2× and 4× MIC of miltefosine, whereas control cells and 0.5× MIC–treated cells did not display increased DNA release over time (**Figure 9A**). A similar kinetic pattern was observed when proteins were measured in the fungal supernatant, in which increasing levels of protein were detected after treatment with 2× and 4× MIC of miltefosine (**Figure 9B**). These results suggest that miltefosine might induce drastic changes in the cellular permeability, resulting in the release of DNA and proteins to the extracellular environment, corroborating with the data observed in **Figures 7** and **8**.

**Figure 10:**
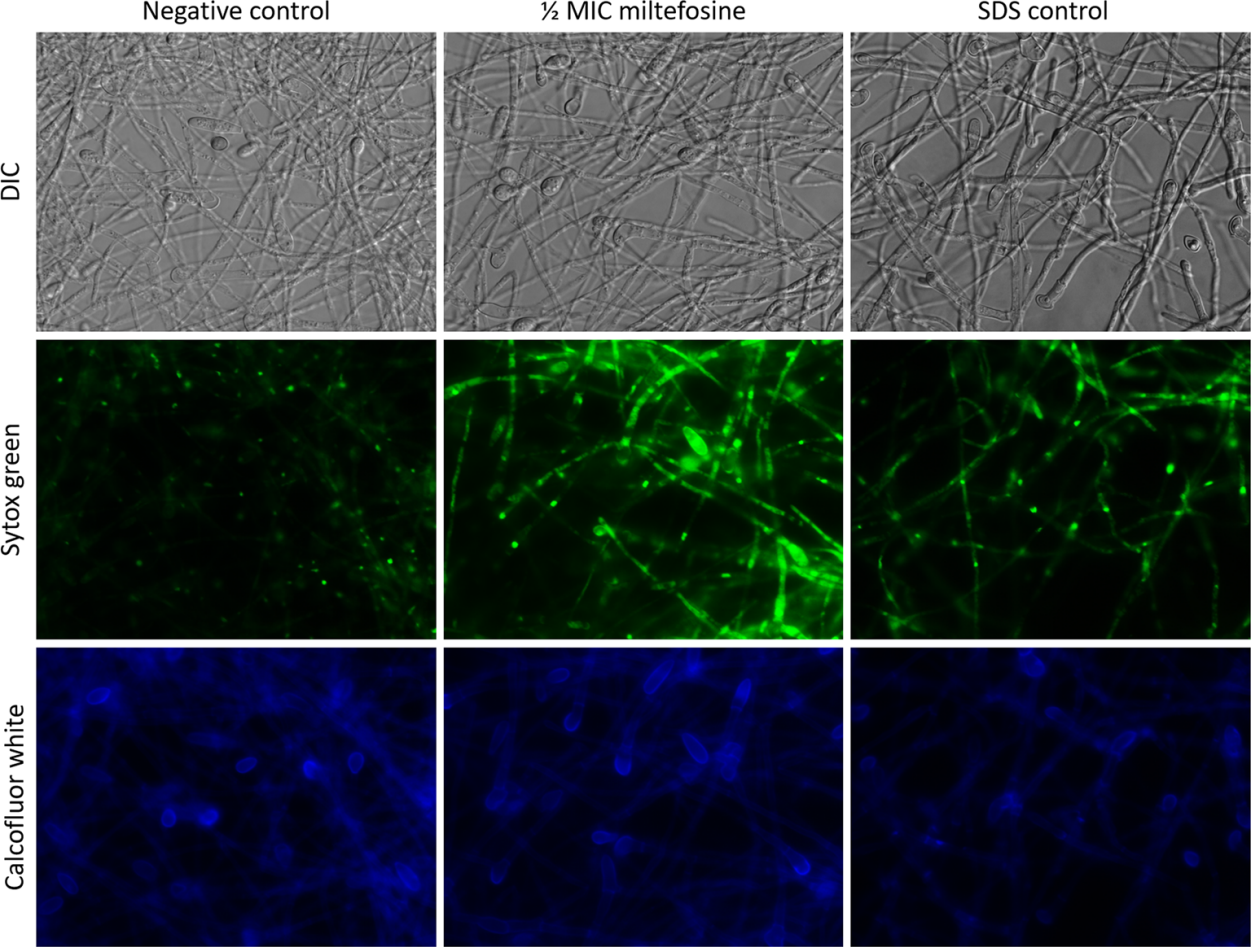
Fluorescence microscopy of *Scedosporium aurantiacum* cells grown for 24 h at 37°C in the absence (negative control) or the presence of 0.5× MIC (2.0 μg/ml) of miltefosine. Sodium dodecyl sulphate (SDS) was used as a positive control of permeable membrane. Cells were stained with Sytox Green, which interacts with nucleic acid of permeable cells, and calcofluor white that stains the fungal cell wall. MIC, minimum inhibitory concentration.

Because there was extracellular DNA and protein observed when cells were treated with miltefosine, we checked the membrane permeability by staining cells with Sytox Green, which stains nucleic acid only in more permeable cells, due to the fact that Sytox Green does not penetrate intact membranes. Compared with non-treated control cells, cells treated with 0.5x MIC of miltefosine seemed to be more fluorescent, suggesting increased membrane permeability (**Figure 10**). A positive control using SDS confirmed that fluorescence increase when the plasma membrane is more permeable. Calcofluor white staining showed that fungal cell wall was not affected: its fluorescence was not changed in treated cells (**Figure 10**).

### Addition of Exogenous GlcCer Decreases Miltefosine Activity

**Figure 11:**
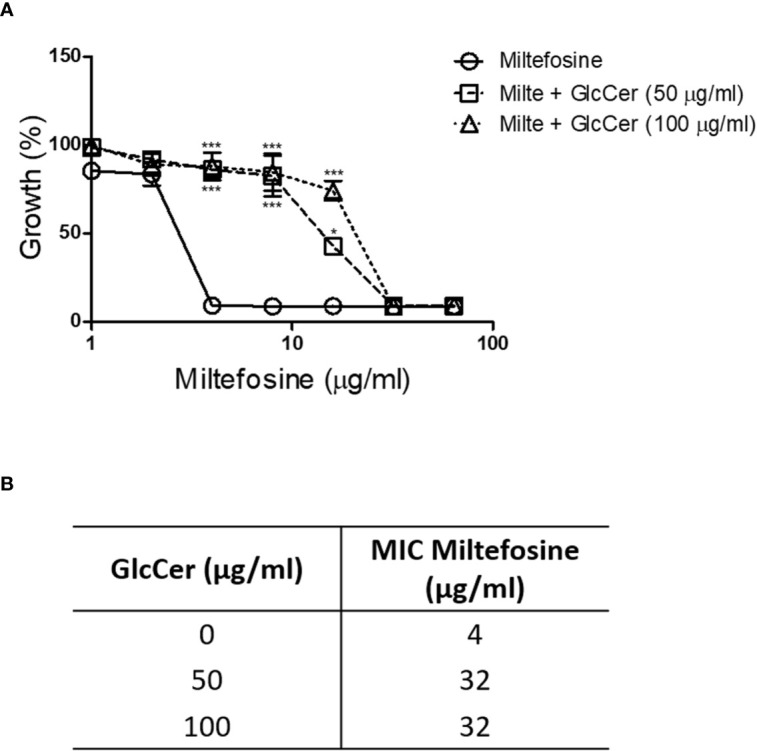
*Scedosporium aurantiacum* susceptibility to miltefosine after exogenous glucosylceramide (GlcCer) addition to the medium. **(A)** *S. aurantiacum* growth at different concentrations of miltefosine. **(B)** The minimum inhibitory concentration of miltefosine when *S. aurantiacum* was grown in the presence of exogenous GlcCer. *p < 0.05, ***p < 0.001.

The presence of GlcCer in the plasma membrane and the cell wall is important to maintain their organisation and structure (Rollin-Pinheiro et al., 2019). When exogenous GlcCer was added to the culture medium, the MIC of miltefosine increased 8 fold (from 4 to 32 µg/ml) (**Figure 11**). This result suggests that miltefosine targets lipids of the *S. aurantiacum* plasma membrane, but more studies are needed to investigate in detail the relationship between miltefosine and fungal lipids.

### Miltefosine Increases the Activity of Inhibitors of Membrane and Cell Wall Constituents

Considering that miltefosine alters the plasma membrane permeability and consequently the cell wall structure of *S. aurantiacum*, we evaluated whether this drug exhibits synergistic effects when combined with inhibitors of membrane or cell wall constituents (**Table 2**). The MIC of miltefosine in combination with fluconazole, caspofungin or myoricin (a sphingolipid inhibitor) was reduced at least 2 fold. After co-incubation with miltefosine, the MIC for fluconazole, caspofungin and myoricin was reduced 2, 15.9 and 16 fold, respectively. According to the FICI interpretation, miltefosine exhibited *in vitro* synergism with caspofungin (FICI ≤ 0.50). These data suggest that combined therapy with miltefosine could be a promising strategy for fungal infections.

**Table 2:** Synergism of miltefosine with fluconazole, caspofungin and myriocin against *Scedosporium aurantiacum*.

	MIC (μg/ml) alone
MLT	4
FLC	25
CAS	25
MYR	16
	MIC (μg/ml) in combination
MLT/FLC	2/12.5
MLT/CAS	1/1.57
MLT/MYR	2/1
	FIC index
MLT–FLC	1.0 (no effect)
MLT–CAS	0.31 (synergic)
MLT–MYR	0.56 (no effect)

MIC, minimum inhibitory concentration; MTL, miltefosine; FLC, fluconazole; CAS, caspofungin; MYR, myriocin.

Experiments were performed in triplicate, in three independent experimental sets, to calculate FICI).

### Miltefosine *In Vitro* Toxicity

**Figure 12:**
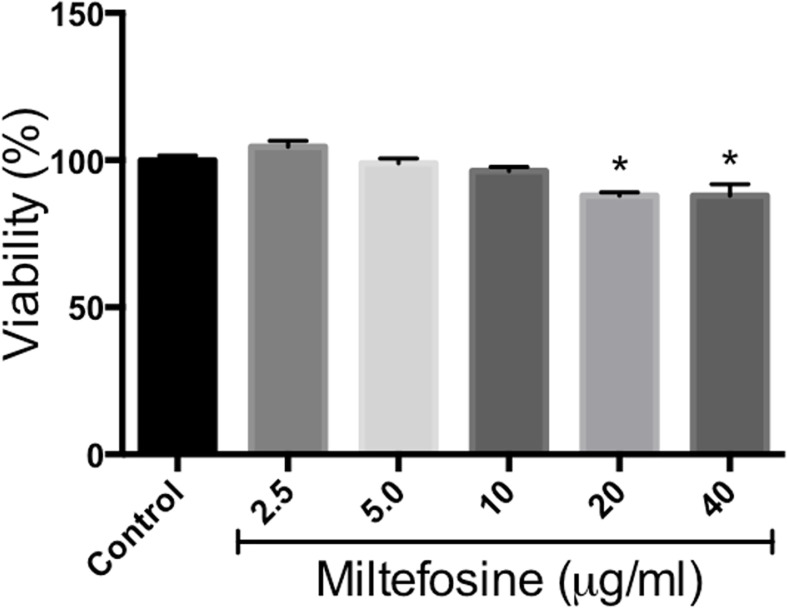
Miltefosine citotoxicity assay. Monolayers of RAW cells were incubated for 24 h in the presence of 2.5, 5, 10, 20 or 40 μg/ml of miltefosine and cell viability was measured by neutral red method. *p < 0.001.

Since miltefosine displayed interesting effects on *S. aurantiacum* cells, as well as present antifungal properties against other species, its toxicity was evaluated using RAW cells. Although statistically significant reduction in cell viability was observed in the presence of 20 and 40 µg/ml of miltefosine, which represents concentrations 5 and 10-fold higher than MIC, cells kept at least 80% of its viability even in the presence of 20 and 40 µg/ml of miltefosine (**Figure 12**). This result suggests that miltefosine is not toxic to RAW cells.

## Discussion

Scedosporiosis is a complex infection that occurs worldwide and can affect not only healthy people, causing superficial infections through a traumatic inoculation of fungal spores, but especially immunocompromised patients, who develop invasive disease after the inhalation of conidia (Cortez et al., 2008). Several conditions have already been associated with scedosporiosis, such as cancer, haematological malignancies, organ transplantation and AIDS (Panackal and Marr, 2004; Mursch et al., 2006; Cortez et al., 2008). For example, a study in 2011 demonstrated that 54.5% of HIV patients presented *Scedosporium* and *Lomentospora* infections, with a 75% mortality rate (Tammer et al., 2011). In addition, *Scedosporium* and *Lomentospora* species are considered the second most frequent fungi associated with pulmonary colonisation in cystic fibrosis patients (Schwarz et al., 2017; Engel et al., 2019).

*Scedosporium* and *Lomentospora* species are commonly resistant to many antifungal drugs currently used in clinical settings, such as amphotericin B, itraconazole, caspofungin, micafungin, isavuconazole and anidulafungin (Lackner et al., 2012). In this context, *S. aurantiacum* has been considered a highly virulent and resistant species (Heath et al., 2009). An *in vitro* study has demonstrated that *S. aurantiacum* displayed 80% mortality in a model of infection using healthy mice (Gilgado et al., 2009).

In the present study, we showed that miltefosine is active against *Scedosporium* and *Lomentospora* at 2–4 µg/ml. Our results corroborate those previously obtained, reporting MICs between 2 and 8 µg/ml (Biswas et al., 2013; Imbert et al., 2014; Compain et al., 2015), but no studies have analysed its effect on the cell biology of *Scedosporium* and *Lomentospora* species. *Scedosporium* and *Lomentospora* growth and viability were affected early by miltefosine, which could be observed between 6 and 12 h of treatment. Similar data have also been observed in *C. neoformans*, *Sporothrix brasiliensis* and *Paracoccidioides* species, whose growth was rapidly impaired by the drug (Borba-Santos et al., 2015; Rossi et al., 2017; Spadari et al., 2018).

It has already been shown that miltefosine displays anti-biofilm activity. In *Candida* species and *Sporothrix schenckii*, mature biofilms were significantly reduced at 16× MIC (64 µg/ml) and 10–50× MIC (40–200 µg/ml), respectively (Vila et al., 2013; Brilhante et al., 2019). Interestingly, *Scedosporium* and *Lomentospora* preformed biofilms were affected in the presence of 4× and 8× MIC of miltefosine, which represents 16 and 32 µg/ml, respectively, indicating that it could be more susceptible compared with other pathogenic fungi. However, further studies are needed to clarify the reduction in the extracellular matrix and also to study in detail the anti-biofilm activity of miltefosine.

To investigate how miltefosine acts on *Scedosporium* and *Lomentospora* cells, we decided to focus on *S. aurantiacum* as a representative species to develop the subsequent analyses. This choice was based on the fact that *S. aurantiacum* has been considered a clinically relevant species due to its high virulence and resistance to antifungals compared with other species (Gilgado et al., 2009; Harun et al., 2010).

The mechanism of action of miltefosine in fungi is still poorly understood. In *Saccharomyces cerevisiae*, miltefosine interacts with COX9, a subunit of the cytochrome *c* oxidase complex in the electron transport chain, leading to disrupted mitochondrial membrane potential and, consequently, cell death due to apoptosis (Zuo et al., 2011). Our results demonstrated that miltefosine decreased the mitochondrial membrane potential and enhanced the ROS levels in *S. aurantiacum*, which are hallmarks of apoptosis (Munoz et al., 2012). Spadari and colleagues have also shown in *C. neoformans* that miltefosine leads to mitochondrial membrane potential reduction and an increase in ROS levels (Spadari et al., 2018), suggesting that apoptosis is a conserved effect of miltefosine in fungi.

Besides causing apoptosis, miltefosine is well known to interact directly with membrane lipids, such as phospholipids, sterols and sphingolipids, in human tumour cells and *Leishmania* spp. (Barratt et al., 2009). This effect involves a specific interaction with membrane microdomains (Heczková and Slotte, 2006). Membrane microdomains, also called lipid rafts, are rich in sterols and sphingolipids that contribute to several important cell processes, such as cellular signalling and regulation of polarised hyphal growth (Fernandes et al., 2016; Rollin-Pinheiro et al., 2019). For these reasons, we used fluorescent staining with filipin, a polyene molecule able to bind ergosterol and commonly used to study fungal lipid rafts. In fluorescence microscopy analysis, when *S. aurantiacum* cells were treated with a sub-inhibitory concentration of miltefosine, the fluorescence intensity was weakened compared with the control, suggesting the presence of disorganised sterol-enriched microdomains in the fungal membrane.

Because miltefosine affected the organisation of lipid rafts, we also evaluated other parameters to study its effect on the *S. aurantiacum* membrane. Miltefosine-treated cells were more susceptible to membrane and cell wall stressors, such as SDS and calcofluor white, suggesting that the plasma membrane loses integrity in the presence of sub-inhibitory concentrations of miltefosine. In addition, release of DNA and protein into the supernatant increased when the cells were grown in the presence of inhibitory concentrations of miltefosine, indicating the occurrence of a leakage of intracellular content. Moreover, Sytox Green staining indicated that miltefosine-treated cells are more permeable compared with non-treated cells. Corroborating these results, scanning electron microscopy analysis revealed that the *S. aurantiacum* surface is significantly modified in the presence of the MIC of miltefosine. An increase in membrane permeability has already been observed in other fungi, such as *C. neoformans*, *Sporothrix brasiliensis*, *Paracoccidioides* spp., *Coccidioides immitis*, and *H. capsulatum* (Borba-Santos et al., 2015; Brilhante et al., 2015; Rossi et al., 2017; Spadari et al., 2018). We suggest that the miltefosine effects on the fungal membrane are similar to those caused by amphotericin B, which increases membrane permeability and, consequently, leakage of intracellular content by interacting with ergosterol and pore formation on the fungal surface (Kathiravan et al., 2012; Mesa-Arango et al., 2012; Spadari et al., 2018).

Exogenous ergosterol addition competes with the target on the *C. neoformans* and *Candida krusei* membrane and increases the miltefosine MIC (Spadari et al., 2018; Wu et al., 2020). Because GlcCer, a sphingolipid well studied in *Scedosporium* and *Lomentospora* species, plays crucial roles in fungal growth and virulence and is a component of lipid rafts, we decided to check whether it could cause the same effect. In the presence of exogenous GlcCer, the miltefosine MIC increase from 4 to 32 µg/ml, suggesting that sphingolipids might also compete with the miltefosine target in the membrane. Besides interacting with lipid rafts, miltefosine has also been described to inhibit sphingolipid synthesis in tumour cells (Jiménez-López et al., 2010). However, it remains to be demonstrated how miltefosine could interact with fungal lipids, as well as which specific lipid could function as a target for the drug.

Due to the effects of miltefosine observed in *S. aurantiacum*, we evaluated the possibility of synergy with the current antifungal drugs used in clinical settings, as well as with the sphingolipid inhibitor myriocin, which also displays antifungal activity and affects the interaction of filipin with membrane lipids in *S. boydii* (Rollin-Pinheiro et al., 2019). Synergism was observed with caspofungin, which targets glucan synthesis. Regarding fluconazole and myriocin, although the calculated FICI index indicated no effect, there was a reduction in the MIC from 25 to 12.5 µg/ml for fluconazole, which targets the synthesis of ergosterol, and from 16 to 1.0 µg/ml for myriocin, which targets the synthesis of sphingolipids. In fact, miltefosine synergism with antifungal drugs, especially regarding *Scedosporium* and *Lomentospora* species, is contradictory. Although some studies have already reported that no interaction is seen between miltefosine and amphotericin B or voriconazole in *L. prolificans*, *S. apiospermum* and *S. aurantiacum* (Cuenca-Estrella et al., 2008; Compain et al., 2015), synergism between miltefosine and voriconazole in some *Aspergillus* strains (Imbert et al., 2014) and between miltefosine and posaconazole or voriconazole in *Fusarium oxysporum*, *L. prolificans* and mucormycete strains has been demonstrated (Biswas et al., 2013). All antifungal drugs used in the present work increase cell permeability by targeting the synthesis of ergosterol (azoles), sphingolipids (myriocin) or glucan (caspofungin). Miltefosine seems to target the plasma membrane, also increasing cell permeability, and although it did not display synergism with all the drugs tested, there was a reduction in the MIC. However, further studies are needed to elucidate the interaction between miltefosine and other antifungal drugs.

Due to the potential of miltefosine as a new therapy for fungal infections in human patients, we evaluated its toxicity using RAW cells. Our results revealed that MIC is not toxic and higher values (5 or 10-fold) keep at least 80% of cell viability. Miltefosine toxicity has also been evaluated in previous studies. Although it has been recently described that the 50% cytotoxic concentration (CC_50_) of miltefosine towards the epithelial cell line LLC-MK2 is 5 µg/ml (Borba-Santos et al., 2015), many other studies indicate that miltefosine toxicity is much higher than the MIC found for *Scedosporium* and *Lomentospora*. Haemolytic activity was observed only at a miltefosine concentration of about 35–40 µg/ml (Widmer et al., 2006; Obando et al., 2007; Valenzuela-Oses et al., 2017), which is almost 10 fold higher than the MIC found for *Scedosporium* and *Lomentospora* planktonic cells in our work. In addition, the cytotoxicity of miltefosine has already been evaluated in a variety of cell lineages, and toxic concentrations were found to be around 38 and 51 µg/ml in MFC (mouse forestomach carcinoma) and SSC (spermatogonial stem cell) cells, respectively (Yu et al., 2021), 21.75 µg/ml in H358 cells (Valenzuela-Oses et al., 2017) and > 25 µg/ml in Vero and HepG2 cells (Ravu et al., 2013). Alginate-nanoencapsulated miltefosine has already been formulated in previous studies and presented no haemolytic or toxic effect in *Galleria mellonella* (Spadari et al., 2019), and the cytotoxic concentrations of micelle-encapsuled miltefosine were found to be 2–3 times higher than the free drug (Valenzuela-Oses et al., 2017). These data indicate that free miltefosine cytotoxicity is about 10 fold higher than the antifungal concentration and that encapsuled version of the drug is also a promising alternative.

*In vivo* studies in *C. neoformans* and *C. albicans* indicate that miltefosine is effective in animal models of fungal infections (Widmer et al., 2006; Vila et al., 2015). It is important to mention that miltefosine is a drug already used in clinical settings, especially for leishmaniosis. Clinical trials have demonstrated that miltefosine is well tolerated by patients and has no toxic effects that could impair its use to treat infectious diseases (Dorlo et al., 2012a). The most common side effects are related to gastrointestinal disorders, such as nausea and diarrhoea, which do not interrupt treatment. Moreover, nephrotoxicity is rare (Sundar et al., 2002; Chrusciak-Talhari et al., 2011; Dorlo et al., 2012b; Sampaio et al., 2019; Wall et al., 2019). In addition, preliminary studies and case reports presenting promising results have also used miltefosine to treat patients with bone and joint infections caused by *L. prolificans*, which demonstrate that it could be applied in the clinic with no significant side effects (Kesson et al., 2009; Quaesaet et al., 2018).

In conclusion, miltefosine induced several changes in *S. aurantiacum* cells at the early stages of treatment, such as plasma membrane disorganisation, loss of membrane integrity, oxidative stress and mitochondrial disturbances. Miltefosine also reduced *Scedosporium* and *Lomentospora* biofilm formation and the viability of preformed biofilm, indicating that it is active against different *Scedosporium* and *Lomentospora* species. Taken together, our results highlight the potential of miltefosine as a therapeutic alternative for fungal infections and clarify its effect in the cell biology of *Scedosporium*, which represents an emerging fungal pathogen.

## Data Availability Statement

The raw data supporting the conclusions of this article will be made available by the authors, without undue reservation.

## Author Contributions

The authors contributed to the work as follows: conceptualisation, RR and YA. Methodology, RR, YA, VR, MX, and LB. Software, RR, YA, VR, MX, and LB. Formal analysis, RR, YA and LB. investigation, RR, YA, VR, MX, and LB. Resources, SR and EB. Data curation, RR, YA, VR, MX, and LB. Writing – original draft preparation, RR and YA. Writing – review and editing, RR, YA, LB, SR, and EB. Supervision, SR and EB. Project administration, SR and EB. Funding acquisition, SR and EB. All authors contributed to the article and approved the submitted version.

## Funding

This study was financed in part by the Coordenação de Aperfeiçoamento de Pessoal de Nível Superior – Brasil (CAPES) – Finance Code 001; Conselho Nacional de Desenvolvimento Científico e Tecnológico (CNPq) and Fundação de Amparo à Pesquisa do estado do Rio de Janeiro (Faperj).

## Conflict of Interest

The authors declare that the research was conducted in the absence of any commercial or financial relationships that could be construed as a potential conflict of interest.

## Publisher’s Note

All claims expressed in this article are solely those of the authors and do not necessarily represent those of their affiliated organizations, or those of the publisher, the editors and the reviewers. Any product that may be evaluated in this article, or claim that may be made by its manufacturer, is not guaranteed or endorsed by the publisher.
